# Life-history traits of a fluorescent *Anopheles arabiensis* genetic sexing strain introgressed into South African genomic background

**DOI:** 10.1186/s12936-022-04276-6

**Published:** 2022-09-05

**Authors:** Nonhlanhla L. Ntoyi, Thabo Mashatola, Jérémy Bouyer, Carina Kraupa, Hamidou Maiga, Wadaka Mamai, Nanwintoum S. Bimbile-Somda, Thomas Wallner, Danilo O. Carvalho, Givemore Munhenga, Hanano Yamada

**Affiliations:** 1 Vector Reference Laboratory, Centre for Emerging Zoonotic and Parasitic Diseases, National Institute for Communicable Diseases, National Health Laboratory Services, Johannesburg, South Africa; 2 Wits Research Institute for Malaria, School of Pathology, MRC Collaborating Centre for Multi-Disciplinary Research on Malaria, Johannesburg, South Africa; 3 Insect Pest Control Laboratory, Joint FAO/IAEA Centre of Nuclear Techniques in Food and Agriculture, Vienna, Austria

**Keywords:** Malaria, Vector control, COPAS

## Abstract

**Background:**

South Africa has set a mandate to eliminate local malaria transmission by 2023. In pursuit of this objective a Sterile Insect Technique programme targeting the main vector *Anopheles arabiensis* is currently under development. Significant progress has been made towards operationalizing the technology. However, one of the main limitations being faced is the absence of an efficient genetic sexing system. This study is an assessment of an *An. arabiensis* (AY-2) strain carrying the full Y chromosome from *Anopheles gambiae*, including a transgenic red fluorescent marker, being introgressed into a South African genetic background as a potential tool for a reliable sexing system.

**Methods:**

Adult, virgin males from the *An. arabiensis* AY-2 strain were outcrossed to virgin females from the South African, Kwazulu-Natal *An. arabiensis* (KWAG strain) over three generations. *Anopheles arabiensis* AY-2 fluorescent males were sorted as first instar larvae (L1) using the Complex Object Parametric Analyzer and Sorter (COPAS) and later screened as pupae to verify the sex. Life history traits of the novel hybrid KWAG-AY2 strain were compared to the original fluorescent AY-2 strain, the South African wild-type KWAG strain and a standard laboratory *An. arabiensis* (Dongola reference strain).

**Results:**

The genetic stability of the sex-linked fluorescent marker and the integrity and high level of sexing efficiency of the system were confirmed. No recombination events in respect to the fluorescent marker were detected over three rounds of introgression crosses. KWAG-AY2 had higher hatch rates and survival of L1 to pupae and L1 to adult than the founding strains. AY-2 showed faster development time of immature stages and larger adult body size, but lower larval survival rates. Adult KWAG males had significantly higher survival rates. There was no significant difference between the strains in fecundity and proportion of males. KWAG-AY2 males performed better than reference strains in flight ability tests.

**Conclusion:**

The life history traits of KWAG-AY2, its rearing efficiency under laboratory conditions, the preservation of the sex-linked fluorescence and perfect sexing efficiency after three rounds of introgression crosses, indicate that it has potential for mass rearing. The potential risks and benefits associated to the use of this strain within the Sterile Insect Technique programme in South Africa are discussed.

## Background

South Africa is one of 21 countries that had set a mandate to eliminate malaria by 2020 as per the World Health Organization’s (WHO) recommendations [1]. This was seen as promising as South Africa’s malaria case numbers have remained constant at around 10,000 cases per annum. However, in 2017, the country experienced a major setback with a spike of almost 20,000 cases, mostly reported in the endemic north-eastern provinces [1]. This called for increased efforts towards vector and parasite surveillance, epidemic preparedness and response, health promotion and vector control as recommended by the WHO. Vector control remains the backbone of malaria strategies in South Africa. Current vector control methods rely on the use of insecticides through indoor residual spraying (IRS) inside dwellings in endemic areas [2]. However, due to increasing insecticide resistance, logistical challenges of implementing IRS under low transmission settings, changing of house structures into modern ones that are unsuitable for IRS application coupled with change in vector behaviour (majority of transmission is now occurring outdoors) [3], calls have been raised for development of new approaches that are more reliable, environment friendly and sustainable to compliment IRS.

Several genetic control methods have been developed aiming at the control of major insect pest and disease vector species, mainly through population suppression strategies, including human disease vectors, such as *Aedes* and *Anopheles* mosquitoes [4]. These include, the sterile insect technique (SIT), the release of insects carrying dominant lethals (RIDL), the incompatible insect technique (IIT), among others [5, 6]. However, the main obstacle towards the implementation of such genetic control population suppression programmes has been the lack of an effective and efficient sex sorting method, particularly for *Anopheles* species, that can guarantee the release of males only [7]. The sterile insect technique (SIT) is one of the proposed approaches because of its species specificity, environmental friendliness and proven success in the suppression or eradication of various major agricultural and animal pests [8, 9]. This technique targets a specific vector population through repeated releases of sterile males of the same species into selected natural environments in order for them to mate with wild females [8, 10]. The result is females laying non-viable eggs. Over time, the number of progeny in target populations is reduced, eventually leading to suppression or even eradication. It is crucial to exclude females from release material as females may contribute to disease transmission through their bites. Males on the contrary cannot contribute to pathogen transmission as they do not bite. It is, therefore, vital that a reliable sex separation system is available prior to deployment of a genetic control method as an additional malaria vector control strategy in South Africa [7, 11].

Some genetic control projects against insect pests and disease vectors have a sex separation system in place, however, none are readily applicable or transferrable between organisms and species [12–15]. With specific reference to mosquito programmes, the main malaria vector in South Africa, *Anopheles arabiensis* remains without an efficient sex separation method [7, 16]. Several methods have been attempted to eliminate females but unfortunately none has been fully developed to be used at an operational level. The available approaches include spiking blood with toxicants to eliminate females and development of a genetic sexing strain (GSS) using classical genetic approaches, reviewed in [7]. To date, the GSS “ANO IPCL1” based on dieldrin resistance developed by Yamada et al. [17], and introgressed into the South African genetic background to create “GMK” by Dandalo et al. [18] has been the only potentially usable genetic sexing strain of *An. arabiensis*. However, the use of dieldrin, a banned insecticide, to achieve sex separation presents challenges. Not only is there the problem of potential harm to the personnel that would need to work with the insecticide on a daily basis, but it has also been found that the dieldrin residues can accumulate in the environment [19]. In addition to this, the strain shows a highly reduced natural fertility rate due to the translocation of the *rdl* (dieldrin resistant) gene, making the strain only 27% fertile [20]. This, coupled with possibilities of recombination, makes it challenging and uneconomic to use in a mass rearing setting [21].

Efforts are being made to develop alternative GSSs using classical genetic approaches and lethal temperature sensitivity mutations and colour-based selectable markers similar to the ones successfully used for the construction of *Ceratitis capitata* VIENNA 8 GSS which is currently used for SIT applications worldwide [8, 22]. Although GSSs are available for some mosquito species, unfortunately, no advanced, ready-to-use sexing systems are currently available for any mosquito species that could be used in an operational programme [23]. A number of alternative sex separation systems are being considered [24–26]. One of the promising methods under development is the sex-specific expression of fluorescent markers at an early developmental stage, ideally embryonic or first instar larval [24, 27, 28]. Such methods can be used to produce a male-only population very early in development, either by conditionally killing the females or by removing them using sex-specific differential expression of fluorescent transgenic markers [24, 29, 30]. Sex separation in early development minimizes rearing costs and facilitates the handling and processing of male-only based releases. In respect to mosquitoes, one of the initial sex-specific strains based on a fluorescent marker was the transgenic sexing line of *Anopheles gambiae.* In this strain, male mosquitoes express enhanced green fluorescent protein (EGFP), under the control of β2-tubulin promoter, thus allowing separation from females at the 4th larval instar stage by using a complex parametric analyser and sorter (COPAS) flow cytometry machine [24]. This system was subsequently improved by Magnussen et al. [31], and the EGFP expression could be detected as early as the 1^st^ instar larval stage. This led to further developments in which the COPAS machine was optimized such that a high-throughput sorting of as much as 20,000 first instar larvae could be achieved in a half hour [32]. Subsequently, Bernardini et al. established a site-specific genetic engineering of the Y chromosome for *An. gambiae* [33] and through this, they developed a hybrid *An. arabiensis* strain carrying the complete Y chromosome of *An. gambiae*, including a red fluorescence protein (DsRed), and potentially other small autosomal genomic regions [34]. This strain, named AY-2, expresses DsRed, in male larvae in the optic lobe and extends down the ventral nerve cord [34].

The use of the one genetic sexing system for several geographic locations is a common practice in population control programmes against agricultural pests. For example, the VIENNA 8 GSS is being used in SIT applications against *Ceratitis capitata* populations worldwide [8]. However, such a practice is not acceptable for the control of mosquito vector populations because of potential inherent differences in vectorial capacity between same species from different geographical locations. If the hybrid transgenic *An. arabiensis* AY-2 strain, which is of foreign (Sudanese (Dongola)) genetic background, is to be considered for the SIT in South Africa, several steps need to be completed, including its introgression into a local genetic background, and basic parameters need to be evaluated. The introgression is necessary to avoid unintended negative impacts such as mating incompatibility and poor competitiveness between the fluorescent strain and the native population. The aim of this study was to start the introgression of the fluorescent marker, which resides on an *An. gambiae* Y chromosome, into a local *An. arabiensis* South African strain and the assessment of the newly developing strain (KWAG-AY2) for its rearing efficiency, fitness, and genetic stability (i.e. sexing reliability), as well as the preservation of the fluorescence intensity and the related sorting efficiency during the introgression process.

## Methods

### Mosquito strains

Four *An. arabiensis* strains, kept at the Insect Pest Control Laboratory (IPCL) of the Joint FAO/IAEA Centre of Nuclear Techniques in Food and Agriculture, Seibersdorf, Austria, were used in this study. The first, Dongola (MRA-856) was acquired from the Northern state of Sudan in 2005 and has since been maintained at the IPCL [20]. The Dongola strain has been used to demonstrate successful mass rearing [35]. The second strain, AY-2 described above was developed from Dongola to express an *An. gambiae* Y-linked red fluorescent protein (DsRed) marker [34]. The third, KWAG was acquired from Kwa-Zulu natal in South Africa and has been under colony since 2005 in the Botha De Meillon Insectary (BDMI), Vector Control Reference Laboratory (VCRL) Johannesburg, South Africa [36]. KWAG originates from Mamfene in Kwa-Zulu Natal, an area earmarked for South Africa’s mosquito SIT pilot studies and is thus a representative of the wild-type population. The fourth, KWAG-AY2, the test strain, was developed by crossing AY-2 males with KWAG females over 3 generations in order to introgress the *An. gambiae* Y chromosome with the DsRed fluorescent marker into the local *An. arabiensis* and, at the same time, achieve about 87.5% replacement of the Sudanese (Dongola) by the South African genomic background.

### Rearing methods

All strains were reared in a climate-controlled room at 27 ± 1 °C and 70% ± 10% RH and a 12:12 h light and dark cycle that includes a 1 h dusk-dawn period. Larvae were reared in a 40 × 80 × 8 cm^3^ tray at a density of  ≥ 1000 larvae in 1 L deionized water and were fed a standard IAEA 1% liquid diet mix of bovine liver powder, Tuna meal and Vitamin mix [35, 37]. Pupae were collected in plastic sample cups of 120 mL capacity and placed in 30 × 30 × 30 cm plastic Bugdorm cages (MegaView Science Co. Ltd., Taichung, Taiwan) and the resultant adults were provided with a constant supply of 5% sucrose solution. Females were provided with defibrinated, defrosted bovine blood twice weekly. Oviposition cups were prepared using filter paper placed on a damp sponge inside a 250 mL round plastic bowl with black lining around the inner perimeter of the bowl.

### Sorting of AY-2 larval populations and introgression of AY-2 into KWAG genetic background

The fluorescence of AY-2 is detectable as early as the first instar under a fluorescent microscope. Initial sorting of AY-2 was done under a fluorescent microscope (Leica MZ16FA, Leica Microsystems GmbH, Wetzlar, Germany), based on the fluorescence on the optic lobe extending down the ventral nerve cord. These were reared to pupation and the sex confirmed as male under a stereomicroscope. KWAG pupae were also gender separated under a stereomicroscope and only females retained. Female pupae were allowed to emerge in individual tubes to confirm sex at adult stage as an extra measure to ensure virginity. AY-2 males were mated with KWAG females for the first round of introgression crosses. The hybrid strain (“KWAG-AY2 (cross 1)”) was then amplified for three consecutive generations before crossing the KWAG-AY2 (1) fluorescent males with KWAG females as described above. The resulting strain was named KWAG-AY2 (2) and is composed respectively of about 25% AY-2 and 75% KWAG genetic background. Progeny resulting from KWAG-AY2 (2) were screened for fluorescence under the fluorescent microscope at varying stages. The hybrid strain (“KWAG-AY2(2)”) was then amplified for three consecutive generations before crossing the KWAG-AY2(2) fluorescent males with KWAG females. The resulting strain was named KWAG-AY2 (3) and is composed respectively of about 12.5% AY-2 and 87.5% KWAG genetic background. As the effects of the introgression progress on the life history traits are not known, it was decided to check the strain’s stability and preservation of the fluorescence, and potentially acquired biological parameters (87.5% introgressed) before continuing with the next 4 rounds of introgression crosses to obtain an *An. arabiensis* strain that contains > 99% South African genetic background with the exception of males that will be carrying an *An. gambiae* Y chromosome since this sex chromosome is strictly paternally inherited. All subsequent life history trait experiments were completed using the KWAG-AY2 (3), henceforth referred to as simply KWAG-AY2. The Leica Application Suite v4.2 was used to capture images of the fluorescent individuals.

L1 larvae need to be unfed in order to be sorted on the large particle flow cytometry machine. Newly hatched, unfed AY-2 larvae, 24–48 h old, were transferred to the reservoir of the large-particles flow cytometry COPAS SELECT™ instrument (Union Biometrica, Inc., Holliston, MA. USA) equipped with a multiline argon laser (488, 514 nm) and a diode laser (670 nm). Analysis and sorting were performed with the Biosort5281 software equipped with a 488 nm filter. The following acquisition parameters: Green PMT 500, Red PMT 600, Delay 8; Width 6, pure mode selection with super drops were used. This is the most stringent setting, meaning that drops that contain two particles (i.e. larvae), of which one is non-fluorescent (females) will be diverted to the “female/waste” receptacle. Post sorting, fluorescent larvae were reared in trays until pupation, pupae sex separated under a stereomicroscope (Leica MZ16FA, Leica Microsystems GmbH, Wetzlar, Germany). Progeny resulting from KWAG-AY2 (3) were screened for fluorescence under the fluorescent microscope at L1 and pupal stage. In other instances, fluorescence was also confirmed in male adults by observing for fluorescence around the eyes.

### Life history traits

#### Egg hatch rates (fertility)

To assess the egg hatching rates of each strain, newly laid eggs (n = 400–500 from each strain) were collected from their respective cages and placed in corresponding clear plastic cups containing 250 mL of deionized water and a drop of larval diet. The plastic cups were lined on the inside with a strip of filter paper to which the eggs adhere. L1 larvae were removed daily. After 72 h, hatched vs. unhatched eggs were counted under a stereomicroscope and comparisons made between the strains. This was repeated three times for each strain with eggs from different cohorts and three oviposition cycles each time.

#### Larval development, pupal emergence, and adult sex ratio

One hundred randomly selected L1 hatchlings from each plastic cup setup during the egg hatch rates experiments were transferred to a 600 mL plastic bowl containing 75 mL of deionized water. Larvae were fed the standard 1% IAEA *Anopheles* diet as follows: 2.5 mL on days 1–3 and 4 mL from day 4 until pupation. Larval trays were inspected once daily for pupae which were then removed and recorded. Once pupation occurred, the number of pupae dead or alive were removed and recorded. The number of days taken for L1 to reach pupation was also recorded. Alive pupae were transferred daily to individual 100 mL sample cups containing distilled water and the cups placed in 17.5 × 17.5 × 17.5 cm bugdorm cage (BugDorm-1H; MegaView, Taichung, Taiwan) for adults to emerge. The number of days taken for L1 and pupae to reach adulthood was also recorded. Similarly, the number of pupae that emerged into adults and the sex ratio of adults were recorded. This was replicated three times, each with three technical repetitions with larvae from different egg batches for each strain.

#### Adult wing lengths

As a proxy for adult size, wing lengths were measured for each strain. Dead adults were collected from cages daily. The right wing was removed from each mosquito, secured onto a sheet of paper using clear tape and the wings were measured by taking images on a microscope camera using Olympus essential v1.9.4 (Olympus Corporation, Shinjuku, Japan). Wings were measured from the distal edge of the alula to the end of the radius vein. The mean length of each wing was recorded, and comparison made between the sexes and strains. These experiments were also repeated three times.

#### Fecundity

Twenty-five newly emerged adult females and males (1:1 ratio) were transferred to 17.5 × 17.5 × 17.5 cm Bugdorm cages and provided with 5% sucrose solution continuously. The adults were allowed to mate for five days after which defibrinated, defrosted bovine blood was provided to each cage for 30–40 min over two consecutive days. All the females were transferred individually into sample cups lined on the inside with a damp filter paper and covered on top with a mesh fabric secured by rubber bands. A 5% sucrose solution was provided through a small strip of filter paper placed on top of each sample cup. Each cup was checked for eggs daily and upon oviposition the total number of eggs laid by each female counted under a stereomicroscope. From this, fecundity was determined from the total number of eggs laid by each female. Three replicates were performed. Females that did not lay any eggs were not included in the eggs per female average.

#### Longevity

Thirty newly emerged males and females (1:1 ratio) from each strain were introduced into 17.5 × 17.5 × 17.5 cm Bugdorm cages, each provided with a 5% sucrose solution continuously. Longevity was monitored daily by removing and counting the dead until no adults remained. A total of two biological repetitions, each with 3 technical replicates were performed for all strains.

#### Flight ability

A flight test device (FTD) developed by the IPCL initially for *Aedes* mosquitoes and modified for *An. arabiensis* was used as described by Culbert et al. [38]. The flight ability test was performed on 100 adult males of AY2, KWAG and KWAG-AY2, with three replicates each. The males were aspirated into the bottom of the flight ability device. A lure was placed on top of the device and a fan was placed over the lure. The adult mosquitoes were allowed to escape for two hours. Escaped males in the chamber and those remaining at the bottom of the FTD were counted as escaped and not escaped respectively, giving an escape rate.

### Statistical analysis

All statistical analyses were performed in R (version 4.0.3) [39] using RStudio (RStudio, Inc. Boston, MA, USA, 2016). Generalized Linear Mixed Models (GLMM, lme4 package) from R were used with the appropriate distribution family. The binomial generalized linear mixed models fit by maximum likelihood (Laplace Approximation) was used, with egg hatch, pupation rate and male flight ability as response variables, strain as fixed effect and the replicate as a random effect.

For the fecundity, A Gaussian linear mixed-effects model was used, with egg number per female as variable, strain as fixed effect and replicate as random effect. Fecundity was analysed with a zero-inflated negative binomial distribution with egg number per female as a response variable, strain (four levels) as fixed factor and replicate as a random factor. Adult wing lengths were analyzed with a Gaussian distribution with strain (four levels) and sex (two levels) as fixed factors and mosquito individuals as a random factor. Data on longevity were expressed as Coxme survival curves. Survival curves were generated using RStudio [39]. A Cox mixed-effects model fit by maximum likelihood with mosquito time to death as response variable, strain (four levels: AY2, KWAG, KWAG-AY2 and DONGOLA) as a fixed factor and replicate as a random factor was used. The full models were checked for overdispersion (using Bolker’s function) [40] and for normality and homogeneity of variances on the residuals [41] for validation. The models were simplified using the stepwise removal of terms, followed by likelihood ratio tests (LRTs) when appropriate. Multiple comparisons using the emmeans function (in package emmeans) [42] were performed between the levels where significant differences were found. A P-value of less than 0.05 was used to indicate statistical significance in all cases.

## Results

### Phenotypic expression of KWAG-AY2

**Fig. 1:**
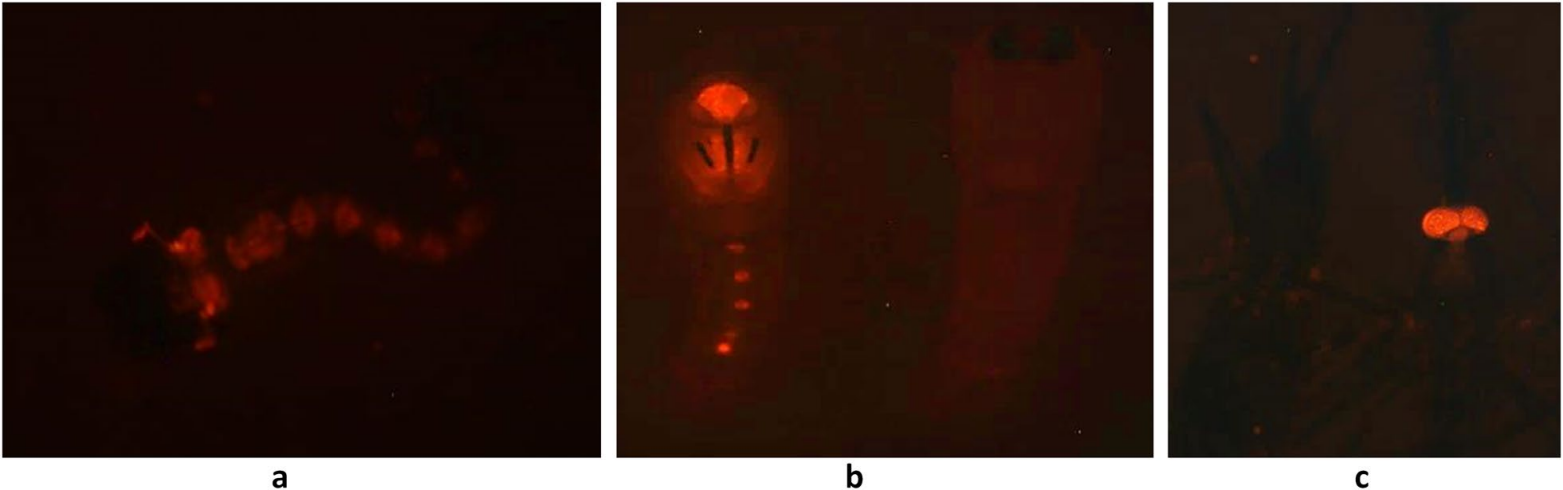
Phenotypic representation of the sorted populations resulting from reciprocal crosses between AY-2 and KWAG under a fluorescent microscope: **a** DsRed fluorescence in L1, **b** Male pupa showing fluorescence on the ocular nerve extending down the nerve cord, left and non-fluorescent female pupa, right, **C** non fluorescent adult female on the left and visible fluorescence in the eyes of adult male, right

The phenotypic representation of AY2 and KWAG–AY2 is shown in Fig. 1. Approximately 400 larvae were sorted both manually and using the COPAS sorter for AY2, 1472 of KWAG-AY2(1) and 779 KWAG-AY2(2) and over 2500 of KWAG-AY2(3). All sorted larvae were kept to adulthood at which the sex was confirmed. Two females were found in pupae or adults sorted as males but these were not fluorescent. The “pure sort” setting was still able to recover ~ 97% of all males. No recombinants (i.e. no non-fluorescent males, or fluorescent females) were found throughout the entire study in all sorted samples (a combined total of 3679 larvae).

### Developmental parameters

**Fig. 2:**
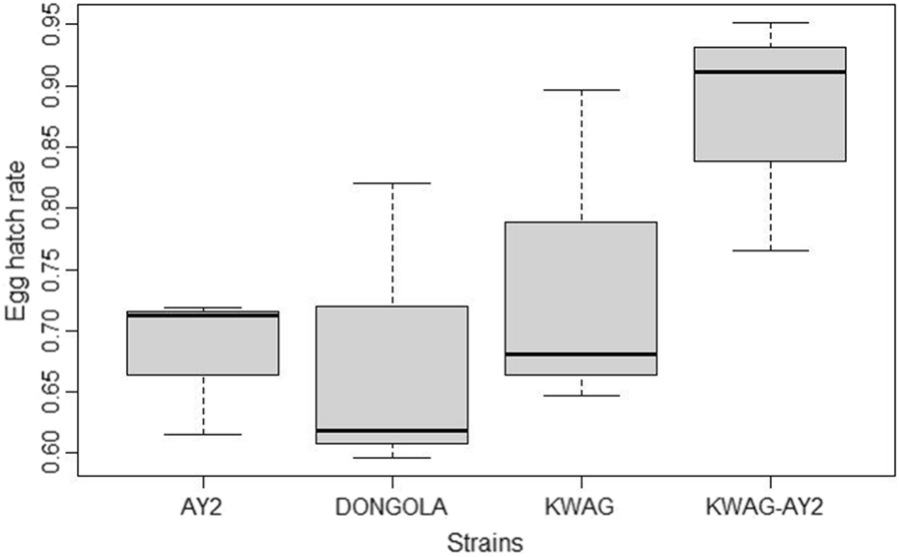
Mean egg hatch rates of AY-2, DONGOLA, KWAG and KWAG-AY2. Each box shows the upper and lower quartiles, the line inside the box (horizontal) represents the median of the sample, while the line outside the box (vertical) represents variability outside the upper and lower quartiles

The mean egg hatch rates were 67% for DONGOLA, 68.3% for AY-2 while KWAG and KWAG-AY2 were 74.2% and 88.1%, respectively (Fig. 2). Statistical interpretation showed a significant difference between the strains (p < 0.05). KWAG hatch rate was significantly higher than AY2 (p = 0.0179) and KWAG-AY2 hatch rate was significantly higher than AY2 (p < 0.001).

#### Larval development and adult sex ratio

Out of the 100 L1 setup for development, the mean proportion surviving to pupal stage was 58.0% for DONGOLA, 26.7% for AY-2, 44.3% for KWAG and 70.3% for KWAG-AY2 (Table 1). Statistical analysis showed significant differences in proportion of L1 surviving to pupal stage between the strains (Table 1 p < 0.001). A Generalized linear mixed model showed that KWAG-AY2 had a significantly higher number of L1s surviving to pupae, whereas AY-2 showed the lowest. The survival rate of L1 to adulthood was significantly different between strains and followed the same pattern as the mean survivorship from L1 to pupae; it was 37.7% for DONGOLA, 19.0% for AY-2, 41.0% for KWAG and 53.3% for KWAG-AY. The proportion of males was as follows: DONGOLA = 60%, AY-2 = 51.7%, KWAG = 49% and KWAG-AY2 = 57.3%. Slight difference in the proportion of males was found only between DONGOLA and KWAG (z = − 1.965, p = 0.05)**.**

**Table 1:** Mean proportion of L1 surviving to pupae, adulthood and proportion of males of four laboratory reared *An. arabiensis* strains. Mean values ± SD, lower–upper 95% CI in brackets

Strain	Proportion surviving from L1 to pupae	Proportion surviving from L1 to adult	Percentage of males
DONGOLA	58.0 ± 23.9 (− 1.4–117.4)^a^	37.7 ± 15.0 (0.3–75.0)^a^	60.0 ± 4.0 (50.1–69.9)^a^
AY-2	26.7 ± 10.4 (0.8–10.4)^b^	19.0 ± 5.6 (5.2–32.8)^b^	51.7 ± 4.9 (39.4–63.9)^a^
KWAG	44.3 ± 15.9 (4.9–83.8)^a^	41.0 ± 26.6 (− 25.1–107.2)^a^	49.0 ± 9.5 (25.3–72.7)^a^
KWAG-AY2	70.3 ± 6.5 (54.2–86.5)^c^	53.3 ± 1.5 (49.5–57.1)^c^	57.3 ± 6.8 (40.4–74.2)^a^

Different superscript letters within columns show that the values were statistically different (P < 0.05)

**Fig. 3:**
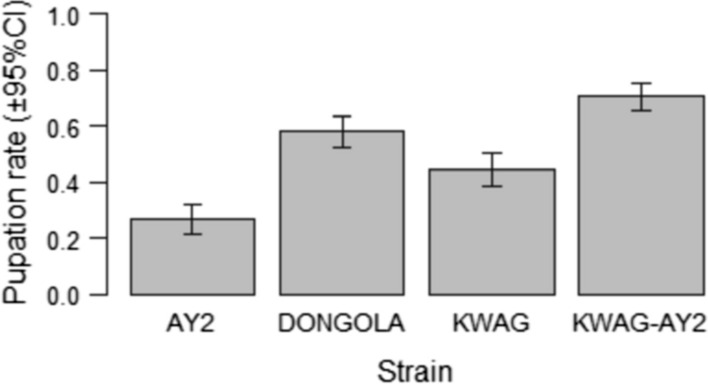
A comparison of the pupation rate of *Anopheles arabiensis* males from the AY2, DONGOLA, KWAG and KWAG-AY2 strains

There was a significant difference in the rate of pupation between AY2 and Dongola, KWAG and KWAG-AY2 (p < 0.001) (Fig. 3).

#### Adult wing lengths

**Fig. 4:**
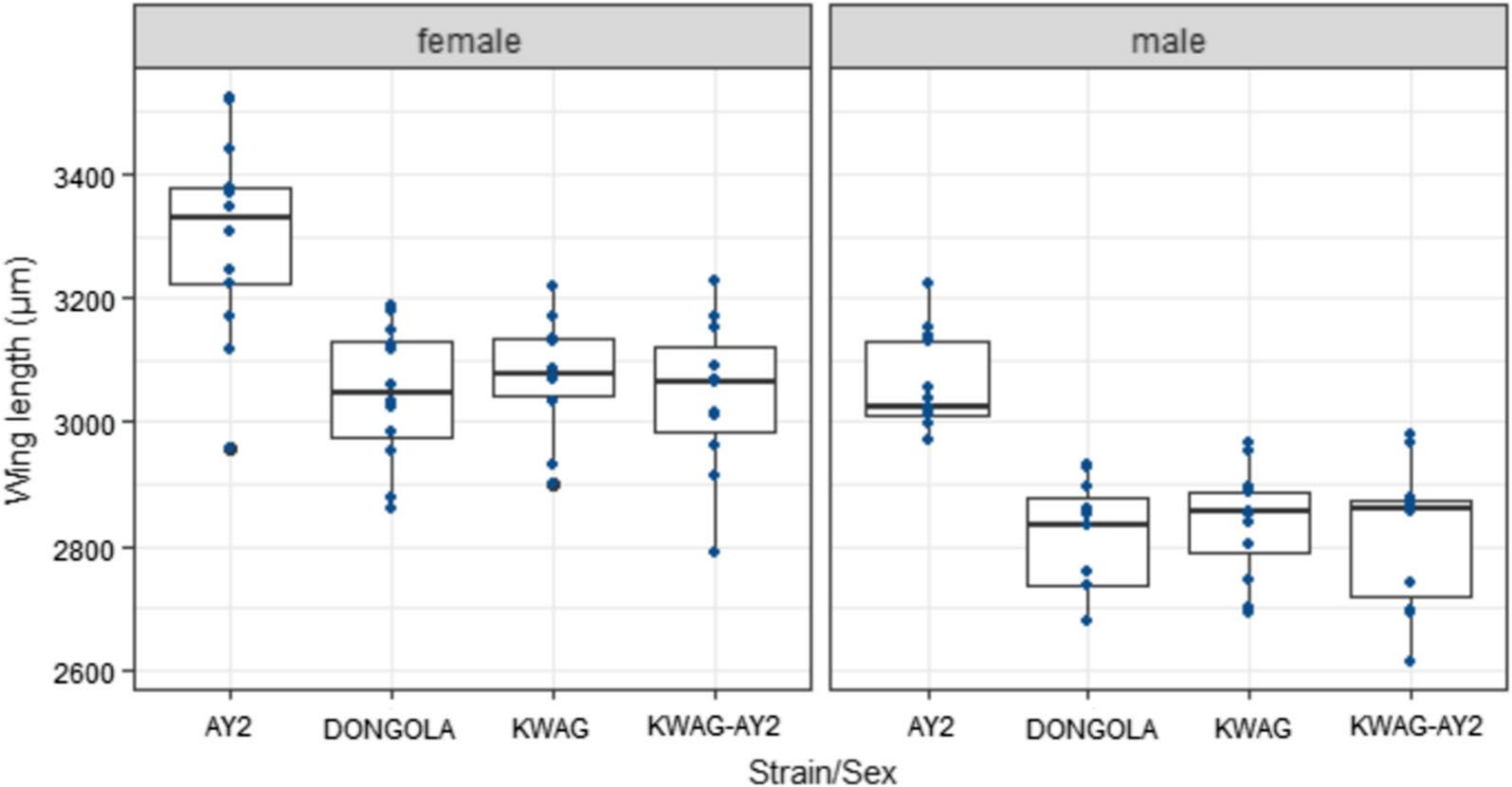
Box plot of wing length measurements for females (left) and males (right) of the 4 comparative strains. Each box shows the upper and lower quartiles, the line inside the box represents the median of the sample, lines extending from the boxes (whiskers) indicate variability outside the upper and lower quartiles and circles indicate outliers

Generally wing length sizes differed between males and females regardless of strain (χ^2^ = 74.1, df = 1, p < 0.001) (Fig. 4). AY-2 females were larger than the males and females of the other strains (χ^2^ = 67.3, df = 3, p < 0.001). Pairwise analysis showed that AY2 males were not significantly different in body size to females of Dongola, KWAG and KWAG-AY2 (p > 0.05).

#### Fecundity

The mean proportion of females that survived in oviposition tubes and laid eggs was 36.0% for Dongola, 41.3% for AY-2, 34.7% for KWAG and 30.7% for KWAG-AY2. Fecundity (total number of eggs laid by each female) of each strain was 76.0 for DONGOLA, 63.3 for AY-2, 58.7 for KWAG and 70.7 for KWAG -AY2 (Table 2). There was no significant difference in fecundity between the strains (p > 0.05).

**Table 2:** Mean number of females that laid eggs and total number of eggs laid per female (fecundity) between four laboratory reared *An. arabiensis* strains. Mean values ± SD, lower–upper 95% CI in brackets

Strain	Mean number of eggs laid per female ± SD (Lower–upper 95% CI)
DONGOLA	76.0 ± 28.5 (5.2–146.8)^a^
AY-2	63.3 ± 15.0 (26.1–100.5)^a^
KWAG	58.7 ± 7.4 (40.4–77.0)^a^
KWAG-AY2	70.7 ± 18.1 (25.6–115.8)^a^

Different superscript letters within columns show that the values were statistically different (P < 0.05)

#### Longevity

**Fig. 5:**
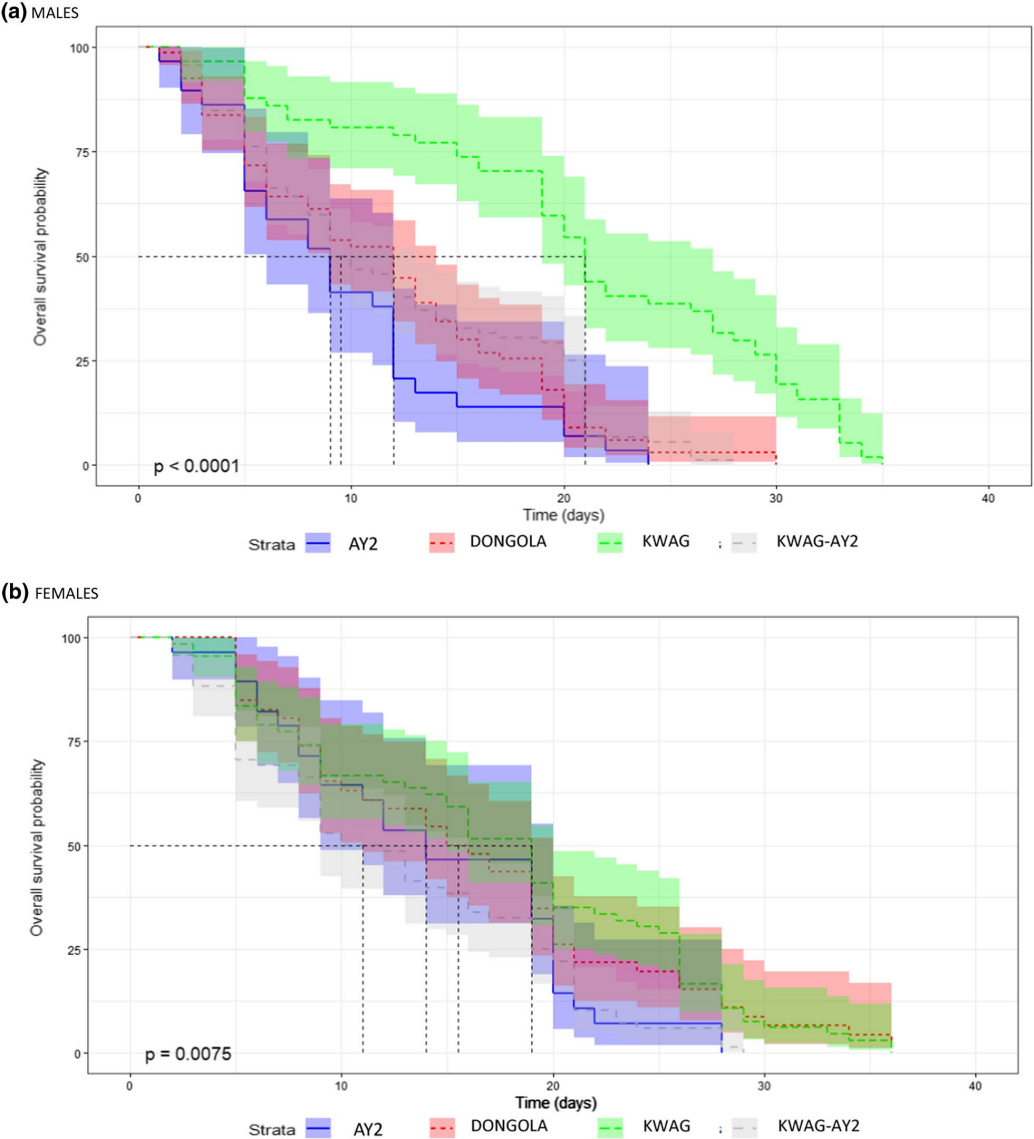
Coxme survival curves for adult males **a** and females **b** of the four *An. arabiensis* laboratory strains

Statistical analysis between the four strains showed significant differences in male survival between the strains (p < 0.0001) as well as in in female survival (p = 0.0075) as shown in the Coxme survival curves in Fig. 5. Coxme analysis revealed that males have a higher risk of death than females. KWAG males had a significantly higher probability of survival than the males of AY2, Dongola and KWAG-AY2 (p < 0.0001).

The median survival time of males in days was as follows: 12.0 for DONGOLA, 9.0 for AY-2, 21.0 for KWAG and 9.0 for KWAG-AY2 while for females it was: 15.0 for DONGOLA, 14.0 for AY-2, 19.0 for KWAG and 10.0 for KWAG-AY2. KWAG females had the highest probability for survival and KWAG-AY2 females had the lowest.

#### Flight ability

**Fig. 6:**
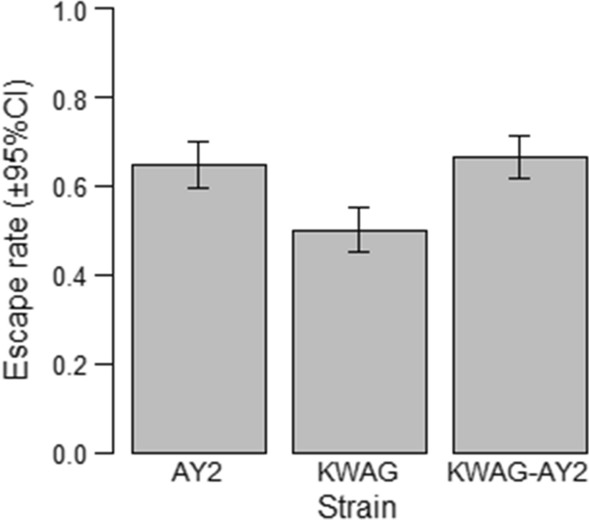
A comparison of the escape rates of *Anopheles arabiensis* AY2, KWAG and KWAG-AY2 males from the flight ability test

The difference in the mean escape rate of the KWAG males compared to KWAG-AY2 and AY2 males was significant (p < 0.0001) (Fig. 6). The mean escape rates were, 64.7% in AY2, 50.1% in KWAG and 66.5% in KWAG-AY2. Pairwise analysis found no significant difference in the escape rates of AY2 and KWAG-AY2 males (p > 0.05).

## Discussion

The aim of this study was to develop a fluorescent sexing strain with a South African genetic background with potential use during the South African sterile insect technique programme. The first part of this work was to assess the strain’s rearing efficiency, its reliability in sex sorting, and to identify any biological factors that may significantly differ from other *An. arabiensis* laboratory strains with a historical record of successful colonisation and the wild type KWAG strain. An *An. arabiensis* KWAG-AY2 sexing strain with a South African genetic background was successfully developed. After three rounds of introgression crosses, the progeny of this strain has approximately 87.5% South African (KWAG) genetic background and with continuous backcrossing a ≥ 99.9% local genetic background will be achieved. The fluorescence and its intensity, and linkage to the male sex were maintained and have still been found to be very stable.

Initial activities involved sorting of AY-2 males, which was mostly done manually under the fluorescent microscope prior to the acquisition of the automated COPAS sorter. No recombination was found from either sorting method in both the original AY-2 strain and the newly established KWAG-AY2 strain. The fluorescence was detectable as early as the first instar larvae both under a fluorescent microscope and the COPAS sorter. It is important to note that larvae need to be unfed in order to be sorted in the large particle flow cytometer, as it seems that larval diet components produce some sort of luminescence that interferes with the laser detection of the fluorescence, producing false positives. Debris either from food, eggshells or legs and wings of adult mosquitoes can cause clogs in the system or cause sorting errors if the particles are of similar size and density to the L1 larvae. However, this should not affect the purity of the males sorted for release. For this reason, larvae were sorted in the first 24–48 h post hatch and before feeding. Young larvae can survive for some time by feeding on the yolk of the eggs after hatching without the addition of food. The female elimination efficiency of the COPAS sorter on KWAG-AY2 was very high (99.97% of over 5000 L1 sorted) as opposed to the 97% elimination efficiency of females reported in GMK [18]. The lack of recombinants in KWAG-AY2, in contrast to the 0.4% recombination rate recorded in ANO IPCL1 and likely related strains, is another advantage towards the applicability of this strain as a sex separation option for the South African SIT programme [18]. The fact that no insecticides are used in the sorting of KWAG-AY2 is another great advantage: the GMK GSS relies on the use of dieldrin for sex separation which poses a constant threat to the colony, staff health, and other non-target insects, and the environment, in which dieldrin residues can accumulate when abundant treated males are released over time [19].

In terms of life history traits, KWAG-AY2 compared favourably against other established *An. arabiensis* laboratory strains in most instances. The life history traits that were assessed included egg hatch rates, larval development, pupal emergence, sex ratio, wing length, adult fecundity, longevity, and flight ability. The egg hatch rate of KWAG-AY2 was similar to that of KWAG and significantly higher than for both AY-2 and Dongola. This is expected due to the genetic similarity between the KWAG-base, and DONGOLA-base strains [34]. In contrast, the GMK GSS, developed from KWAG females and males of the original GSS ANO IPCL1, has a high natural sterility due to the chromosomal translocation and possibly exacerbated by introgression into the local genomic background, giving low egg hatch rates of 19.2% [18]. This low productivity makes the GMK GSS uneconomic for mass rearing as a male recovery rate of less than 10% of any egg batch produced during mass rearing renders the production inefficient.

KWAG-AY2 had a significantly higher proportion of L1 surviving to pupae as compared to AY-2, and slightly higher, but not statistically different to KWAG and Dongola. Dongola provides the standard for mass rearing; thus, this characteristic bodes well for the chances of KWAG-AY2 performing well in a mass rearing setting. Each strain was fed the same amount of food and larval density was the same at experimental set up. The optimal feeding regime for Dongola as described in the standardized mass rearing guidelines, may not be ideal for the other tested strains. The rearing water in the AY-2 containers was clear, whereas the water became cloudy in the 2 KWAG-based strains. This suggests that AY-2 requires more diet than the other strains, while the KWAG strains might require less. The early mortality and overall low survival rate in the AY-2 larvae may be due to starvation of some of the larvae and necrophagy by those remaining (as no dead larvae were seen). This led to a reduced rearing density, and thus more food for the survivors, resulting in faster development times and larger adult body size. Adjusting diet amounts to food per larva would improve the experimental design to compare body size. Also, a feeding regime that yields higher survival of the transgenic AY2 and KWAG-AY2 strains needs to be further investigated as overall the survival of larvae for all the strains used in this comparison were low. However, the importance lies in the result that the KWAG-AY2 adults did not differ in size to the KWAG *wild-type* adults, as compatible body size is important in mating preferences [43].

Marois et al. [32] suggested that when mass rearing transgenic insects, fitness costs may arise by inbreeding, as is seen in most colonized and artificially reared insects [44, 45]. To counteract this and increase the gene pool, it is necessary to apply good colonization and maintenance principles, allowing genetic diversity [23, 46].

It is important to consider the different developmental stage characteristics as they have a bearing on the productivity and quality of males to be released. The faster larval developmental time shown by KWAG-AY2 as compared to the other strains is comparable to results from other studies such as 6.9–7.4 days in GSS ANO IPCL compared to Dongola (7.5–7.8 days) [20]. In this study, it took Dongola on average of 8 days to pupation and 10 days to reach adult emergence. Pupation of KWAG-AY2 took on average 8 days and 9 days to adult emergence compared to GMK which took an average of 9 days to develop to pupae and 12 days to adult emergence.

The percentage of females that laid eggs was lower than what is usually observed in *Aedes* spp. *Aedes* mosquitoes are not averse to being isolated in small containers for individual oviposition, presumably because such an environment is close to that encountered by these species in nature. In contrast, *Anopheles* are generally more fragile, and do not survive well when confined in small spaces and are often observed to prefer withholding eggs rather than laying them in an “unsuitable” place (personal observations). The low fecundity levels recorded is therefore not unusual and was expected in all the four strains, and those that survived and did not lay eggs may be the result of not all females having blood fed or mated, or females withholding eggs. KWAG-AY2 showed high fecundity, and high natural fertility (hatch rate). These characteristics imply that there are no major limitations in the mass rearing of this strain. Taking only those individuals into account that laid eggs, the differences in numbers of eggs laid/female between the four strains were not significant. Under mass rearing conditions, Mamai et al*.* [47] demonstrated that *Anopheles* females laid an average of 40 eggs per female when 15 000 pupae were placed in mass rearing cages, however, the eggs were quantified *en masse*, and were divided by the initial number of female pupae placed in the cage. In another study, Oka et al*.* [48] found female *Anopheles* laid an average of 52 eggs per female in a substrate preference experiment. Mosquito fecundity is variable and ranges between 50 and 200 eggs per oviposition have been recorded [49, 50]. Egg counts in this current study are comparable with other *Anopheles* egg counts considering the scale of the experiment.

Longevity of adult males is important for SIT mass rearing and release. Male median survival time was longer for KWAG males than the other strains. KWAG-AY2 (9 days) was not significantly different from Dongola and AY-2 (9 and 12 days respectively). For field releases, males need to survive long enough to find suitable mates and copulate with as many females as possible. Further tests need to be conducted to determine how irradiation and release in semi-field conditions may affect longevity and mating capacity of KWAG-AY2 as compared to the KWAG strain.

The use of flight test cylinders as a routine method of quality control in insect rearing has been shown to effectively demonstrate the quality of flying insects [38, 51]. Released laboratory males are required to find and mate with wild females and this is dependent on their ability to fly. Flight test devices have been adapted for *Anopheles* and used to demonstrate the effects of different treatments on male flight ability, which was correlated to mating propensity and survival [38]. In this study, KWAG-AY2 had an escape rate that was significantly higher than KWAG but similar to the AY2 strain. Further studies of flight ability of KWAG-AY2 after irradiation need to be assessed as males earmarked for release need to be sterile. KWAG-AY2 has performed well when compared to reference strains in terms of egg hatch rates, larval survival and adult size, fecundity and flight ability, compared to the other reference strains and thus is promising for its mass rearing potential. The relative faster developmental rate for KWAG-AY2 observed in this study is also a favourable characteristic from a mass rearing perspective as this could reduce operational costs. Taking all of the rearing parameters into account (fecundity × egg hatch rate × survival to adulthood), the rate of increase was found to be 19.2, 8.2, 17.9 and 33.0 for the *An. arabiensis* strains Dongola, AY2, KWAG and KWAG-AY2 respectively. The success of the KWAG-AY2 strain may have been contributed by heterosis, by crossing the KWAG and AY2 strains.

The use of strains with sex-linked fluorescence coupled with COPAS sorting may represent a valid option to large-scale male-only releases for the South African SIT programme. The sorting speed of COPAS would allow the accurate sorting of almost 2 million male mosquitoes a week with 8 h shifts per day [32]. The use of COPAS for sorting fluorescent mosquito larvae has proven to be a highly efficient and reliable method for *Anopheles* sex separation [32].

## Conclusion

Realistic conclusions on the potential use of KWAG-AY2 can only be drawn after the full introgression of the local genomic background, the evaluation of its rearing efficiency at large scale, irradiation dose–response of the strain, the effect of irradiation on male mating competitiveness, mating compatibility with the wild-type KWAG strain, and then ultimately mark release recapture (MRR) studies in open field conditions to complete the evaluation phases to consider this new strain for larger scale suppression programmes [11, 52, 53]. In addition, a risk assessment analysis is required to address any concerns related to the potential release of this transgenic and hybrid *An. arabiensis* strain in ecosystems as well as to obtain the necessary regulatory approvals for open field releases [26]. Finally, the release of irradiated fluorescent males would allow their easy monitoring in the environment and checking for the absence of introgression of the wild target population of *Anopheles*. However, the development of non-transgenic strains by linking a marker on the Y chromosome using genome editing approaches would be desirable in the future.

## Data Availability

Data and material can be made available upon reasonable request.
